# Study on the limit of detection in MZI-based biosensor systems

**DOI:** 10.1038/s41598-019-42305-8

**Published:** 2019-04-08

**Authors:** Daan Martens, Peter Bienstman

**Affiliations:** Photonics Research Group, INTEC, Ghent University - imec, Ghent, 9000 Belgium

## Abstract

Mach-Zehnder interferometers are integrated photonic sensors that have yielded excellent detection limits down to 10^−7^ RIU. They are of particular interest due to their large design freedom, allowing for example application in promising point-of-care compatible read-out schemes. The attainable detection limit of such sensors can interact with the sensor design in different ways, depending on the dominant origin of noise which can either be influencing a single sensor arm, both sensor arms or can be unrelated to the sensor itself. In this work, the interaction of these three noise regimes with the sensor design is examined. The regimes are combined into a framework that predicts the limit of detection as a function of sensor design. A set of experimental results confirms the validity of this obtained theoretical framework. This analysis provides a blueprint for optimization of MZI photonic sensors under any combination of read-out method and measurement circumstances.

## Introduction

Label-free integrated evanescent field biosensors can provide qualitative as well as quantitative measurements in real-time, rendering them excellent candidates for point-of-care diagnostic applications. In research, various types of such sensors have been developed, including ring resonators, photonic crystals and various types of interferometric sensors^1–7^. This plethora of research output is in stark contrast with the low number of available commercial applications, which furthermore are typically limited to the research market or clinical laboratories^8–10^. An important barrier for further commercial implementation of these sensor types is the typically high instrumentation cost, as expensive components are typically required for optical readout (a tunable laser or an optical spectrum analyzer) and/or alignment (microprecision stages). These factors limit the attainable application range to the aforementioned higher end of the market, whereas the low chip costs renders these sensor types inherently well suited for the lower, point-of-care end of the market, e.g. self-tests or GP office based tests.

In^11,12^, a sensing scheme was proposed that can overcome these instrumentation barriers. It consists of an integrated photonic sensor combined with an on-chip spectral filter, a circuit that can be interrogated through an LED and a CMOS camera. When grating couplers are used at the input^13^ in combination with flood illumination^14^, the required alignment precision is greatly reduced, to a point where active alignment is no longer required. This low-cost interrogation scheme is therefore fully compatible with point-of-care applications.

As a sensor for this circuit, the Mach-Zehnder interferometer is an optimal choice as its sensitivity and spectral period can be independently adjusted^12^. This is particularly relevant as the spectral period needs to be adapted to the technological limitations of the integrated spectral filters^15^. In^12^, this sensor circuit was theoretically and experimentally examined. This fully point-of-care compatible scheme yielded detection limits similar to those of other integrated photonic sensors with more expensive read-outs^12^.

In this work we will further explore the possibilities and limitations of MZI-based sensor systems. In^12^ we examined how certain intensities of noise on the sampled sensor spectrum translate to minimum detectable wavelength shifts. In this work we will take a slightly different approach, looking at the final limit of detection as a function of various possible dominant noise sources. We will examine how, in various noise regimes, MZI sensor design can (or cannot) influence the detection limit, and confirm this through experimental results. This will result in universal guidelines on MZI sensor design optimization in the given sensing scheme and beyond.

## Mach-Zehnder interferometers as photonic integrated sensors

The Mach-Zehnder interferometer (MZI) is a specific type of interferometric sensor, consisting of a photonic waveguide split in two, typically symmetrically. After propagation through the arms (which can have different lengths), the light is recombined, interfering destructively or constructively depending on the accumulated phase difference. As such, the accumulation of phase is translated into a change in the transmitted amplitude which varies as a function of wavelength. In photonic sensing, one of these arms is typically exposed to the analyte, while the other is isolated from it, either through physical isolation from the sample liquid or through a lack of receptor molecules present on the waveguides. As such, the attained phase shift per change in environmental refractive index (through binding events or bulk index change) can be made arbitrarily large by increasing the length of the sensing arm, whilst adjusting the length of the reference arm to maintain desirable spectral characteristics. The 2*π* phase ambiguity present in these systems is also not an issue: in most interrogation mechanisms, including the one discussed, the frequency of measurement is high compared to the speed of the biological phenomena, which allows to continuously monitor the phase.

In practical devices, the phase is often not directly tracked, but rather through the peak wavelength. In that case, the sensitivity is expressed in nm/RIU and smallest detectable shift is expressed as the smallest detectable peak wavelength shift. For the remainder of this work, we will speak in terms of phases rather than peak wavelength, but they are equivalent, as a phase shift of 2*π* corresponds to a peak shift of a full spectral period.

The freely tunable sensitivity does of course not imply that an arbitrarily small quantity of analyte can be detected by simply increasing the sensing arm length. The limit of detection is influenced both by the sensitivity as well as the smallest detectable phase shift. This smallest detectable shift can also be influenced by the sensing arm length in various less trivial ways.

## Detection limit of Mach-Zehnder interferometer photonic sensor systems

In^12^, an extensive theoretical study of the MZI as refractive index sensor was carried out. In a typical sensing device, one arm is exposed to the analyte and the other arm is isolated from it. In that case, the relevant sensitivity is found by calculating the phase shift Δ$\varphi$ caused by an effective refractive index change ${\rm{\Delta }}{n}_{eff,s}$ in the sensing arm. The sensitivity is found to be:

1 $${S}_{1arm}=\frac{{\rm{\Delta }}\varphi }{{\rm{\Delta }}{n}_{eff,s}}=\frac{\pm 2\pi \nu {L}_{s}}{c}$$

Here, *ν* represents the frequency and *c* is the speed of light. The sensitivity is positive if ${n}_{eff,s}{L}_{s}$ is larger than ${n}_{eff,r}{L}_{r}$, subscript *s* and *r* referring to sensing and reference arm respectively. As assessed before, this sensitivity can be tuned freely through increase of the arm length. However, the true figure of merit for sensing is the limit of detection, given by

2 $${\rm{detection}}\,{\rm{limit}}=\frac{{\rm{minimum}}\,{\rm{detectable}}\,{\rm{phase}}\,{\rm{shift}}}{{\rm{sensitivity}}}$$

The minimum detectable phase shift (mdps) is defined as three times the standard deviation on the detected phase during a period of time when no external change is being applied. To be able to improve on the detection limit, it is therefore vital to understand the determining factor for this minimum detectable phase shift. This mdps is influenced by a large number of factors, each of which could be dominant. These factors can be divided in three categories through their relation with the MZI sensor as each of these will result in a different LOD regime. The determining factor can either be unrelated to the sensor (regime a), related to a single arm (regime b), or related to both arms (regime c). In the following sections, these regimes will be analyzed separately before combining them into a single framework.

In regime a, the most simple possibility, the factor that determines the mdps is unrelated to the sensor itself, e.g. the noise on the detection mechanism (like camera noise). In that case, from a sensor design point of view, the minimum detectable wavelength shift can be seen as a constant 3*σ*_*a*_. Note that this final 3*σ*_*a*_ is a function of the whole read-out system, including for example the number of filter channels in the (on-chip) spectrometer. In this work, we are analyzing only the dependence on MZI sensor design, and assume a ‘fixed’ read-out mechanism. With such a *σ*_*a*_, the limit of detection is given by:

3 $${{\rm{LOD}}}_{{\rm{a}}}=\frac{3c{\sigma }_{a}}{2\pi \nu {L}_{s}}$$

As such, improvement in the detection limit can be achieved simply through lengthening the sensor, as it scales with 1/*L*_*s*_.

In regime b, the other extreme, the dominant noise source is related only to the sensing arm, and not to the reference arm. An example is the noise caused by inhomogeneity of the sample liquid, to which the typically clad reference arm is not exposed. In that case, the effective refractive index of the sensing arm will be normally distributed with a certain standard deviation *σ*_*b*_.

4 $${\rm{\Delta }}{n}_{eff,s}\sim {\mathscr{N}}(0,{\sigma }_{b}^{2})$$

Note that *σ*_*b*_ is the value registered by the sensing system, and can therefore be larger than the actual standard deviation on the effective refractive index, due to the imperfection of the system. From this and Eq. (1), the distribution of the phase in the described case is found as:

5 $${\rm{\Delta }}\varphi \sim \frac{2\pi \nu {L}_{s}{\mathscr{N}}(0,{\sigma }_{b}^{2})}{c}$$

As such, the standard deviation on the phase as a whole is given by $2\pi \nu {L}_{s}{\sigma }_{b}/c$, which means that the LOD is given by:

6 $${{\rm{LOD}}}_{{\rm{b}}}=\frac{6\pi \nu {L}_{s}{\sigma }_{b}}{2\pi \nu {L}_{s}}=3{\sigma }_{b}$$

In regime b, the limit of detection is therefore not influenced at all by the sensing length, as the change in numerator and denominator in Eq. (2) cancel each other out. As such, in this case the limit of detection cannot be improved through adapting the sensor design, and this regime potentially results in a fundamental limit of detection (if the source of noise cannot be addressed elsewise). The more unlikely case where a noise source would only affect the reference arm rather than the sensing arm can be described in an identical manner, of course upon substitution of the properties of the sensing arm by those of the reference arm.

Regime c, the most complex case, is a dominant noise source that influences both MZI arms, for example homogeneous temperature fluctuations. In case the reference arm is not clad and the reference arm is also exposed to the sample liquid (but has no receptors), inhomogeneity of the sample liquid also follows this regime. This type of noise will affect both arms, but not completely identically, due to their potentially different cladding, different geometry or possibly fabrication variations. Taking the case of homogeneous temperature fluctuations, a normally distributed temperature with standard deviation *σ*_*c*_ will result in the following distributions of the effective refractive indices of both arms:

7 $${\rm{\Delta }}{n}_{eff,s}\sim {C}_{sT}{\mathscr{N}}(0,{\sigma }_{c}^{2})$$

8 $${\rm{\Delta }}{n}_{eff,r}\sim {C}_{rT}{\mathscr{N}}(0,{\sigma }_{c}^{2})$$

here, *C*_*sT*_ en *C*_*rT*_ are the temperature constants of the respective waveguides. With Eqs (7) and (8), the phase distribution becomes:

9 $${\rm{\Delta }}\varphi \sim \frac{2\pi \nu }{c}({C}_{rT}({L}_{s}-{L}_{r})+{L}_{s}({C}_{sT}-{C}_{rT})){\mathscr{N}}(0,{\sigma }_{c}^{2})$$

Here, the term in *L*_*s*_ − *L*_*r*_ represents an effect that is identical for both arms, whereas the term in *L*_*s*_ accounts for the difference between both arms. In case the difference between *C*_*sT*_ and *C*_*rT*_ is large, the first term becomes negligible (except for typically irrelevant low sensing arm lengths) and regime c becomes equivalent to regime b, a phenomenon only affecting one arm. Other noise sources adhering to regime c will yield the same behavior, and can be found by substituting the temperature constants of the waveguides by the corresponding waveguide properties.

Equation (9) in combination with Eq. (1) yields a limit of detection within regime c given by

10 $$LO{D}_{c}=3{\sigma }_{c}{C}_{rT}|\frac{{C}_{sT}}{{C}_{rT}}-\frac{{L}_{r}}{{L}_{s}}|$$

where *σ*_*c*_ is independent of the MZI sensor design. This means that the only way to improve the limit of detection through the MZI is through $|{C}_{sT}/{C}_{rT}-{L}_{r}/{L}_{s}|$. This initially suggest adapting the sensor and reference arm lengths for this term to equal 0 can easily result in infinitely small limits of detection. However, most, if not all, practical systems are limited in bandwidth as well as spectral resolution. Therefore, as the arm lengths directly determine the spectral period, they need to adhere to certain limitations. Taking a more practical approach, we look at the situation where we only consider sensors of a given spectral period. In an MZI sensor, the spectral period in the frequency regime *p*_*ν*_ is given by:

11 $${p}_{\nu }=\frac{c}{|{n}_{g,s}{L}_{s}-{n}_{g,r}{L}_{r}|}$$

where *n*_*g*,*s*_ and *n*_*g*,*r*_ are the respective group indices of sensing and reference arms. By extracting *L*_*r*_ as a function of *L*_*s*_ from Eq. (11) and substituting it in Eq. (10), we get the following expressing for the LOD:

12 $$LO{D}_{c}=3{\sigma }_{c}{C}_{rT}|\frac{{C}_{sT}}{{C}_{rT}}-\frac{{n}_{g,s}}{{n}_{g,r}}\pm \frac{c}{{p}_{\nu }{n}_{g,r}{L}_{s}}|$$

where a plus sign corresponds to a situation where ${L}_{s}{n}_{g,s}-{L}_{r}{n}_{g,r} > 0$ and a minus sign corresponds to ${L}_{s}{n}_{g,s}-{L}_{r}{n}_{g,r} < 0$. This means that, if both ${L}_{s}{n}_{g,s}-{L}_{r}{n}_{g,r}$ and ${n}_{g,r}{C}_{sT}/{C}_{rT}-{n}_{g,s}$ are positive or are both negative, corresponding to $({L}_{r}{n}_{g,r}-{L}_{s}{n}_{g,s})\,({n}_{g,r}{C}_{sT}/{C}_{rT}-{n}_{g,s}) > 0$, we get a hyperbole, asymptotically approaching 3*σ*_*c*_$|{C}_{sT}/{C}_{rT}-{n}_{g,s}/{n}_{g,r}|$. In the alternative case, corresponding to $({L}_{r}{n}_{g,r}-{L}_{s}{n}_{g,s})\,({n}_{g,r}{C}_{sT}/{C}_{rT}-{n}_{g,s}) < 0$, a more complex situation arises. In this case, The LOD becomes minimal for a certain length. This is explained because, to achieve the required period with the required indices, at this specific point sensing and reference arm will be of the following length: ${L}_{s}={L}_{r}{C}_{rT}/{C}_{sT}=c/(p|{n}_{g,s}-{n}_{g,r}{C}_{rT}/{C}_{sT}|)$, meaning that a noise source which affects both arms homogeneously by definition will have no effect. A peculiar consequence of this is that a longer MZI will actually have a worse limit of detection. This local minimum can therefore be a potentially interesting point to target, especially for MZIs with arms with high index contrast, where it will occur at relatively low arm lengths. When targeting this local minimum in case the dominant factor is not yet identified or the properties of the waveguide have not been experimentally determined, an initial design with equal lengths is a good approximation, as ${C}_{rT}/{C}_{sT}$ will typically be close to one.

**Figure 1:**
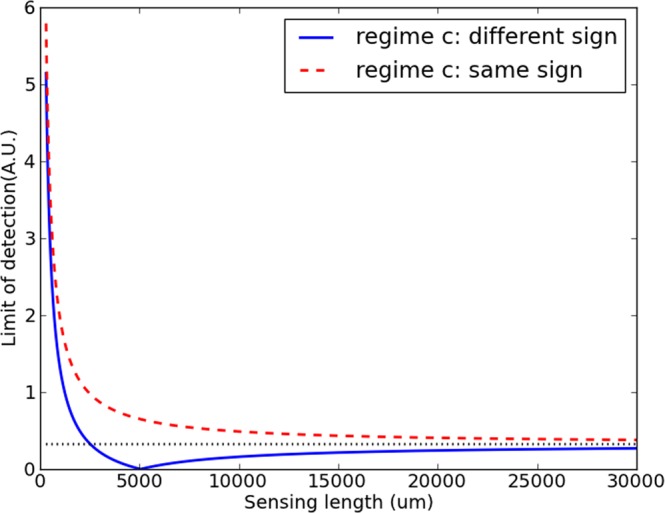
Detection limit as a function of sensing arm length for a series of MZIs with the same spectral period in case the dominant noise source applies homogeneously to both arms (situation c).

These phenomena are illustrated in Fig. 1, where the red dashed curve represents the situation where ${L}_{r}{n}_{g,r}-{L}_{s}{n}_{g,s}$ and ${n}_{g,r}{C}_{sT}/{C}_{rT}-{n}_{g,s}$ are of the same sign, for the blue continuous curve they are of opposing sign. Only in the latter curve, a local minimum emerges. These simulation were based on a realistic MZI made up of silicon nitride waveguides of 700 nm by 150 nm, designed for single mode operation around 850 nm. The claddings of the arms were water (sensing arm) and silicon oxide (reference arm), and their respective group indices were set at simulated values of 1.8674 and 1.8878. Their temperature constants *C*_*rT*_ and *C*_*sT*_ were assumed equal. The spectral period of the sensors was set at 7 nm, rending them measurable with spectral filters with a channel spacing of 1 nm, which are technically feasible in this wavelength range^15^. The *σ*_*c*_ of 10.0 was chosen arbitrarily.

It is also noteworthy that the limit of detection at very high sensor lengths becomes $3{\sigma }_{c}|{C}_{sT}/{C}_{rT}-{n}_{g,s}/{n}_{g,r}|$. While no longer dependent on the arm lengths, this value can also be influenced: the easiest way to do this is designing the arm waveguides as similar as possible, as fully identical waveguides would bring this limit to 0. The possibility of this of course depends on the available design freedom. This implies that a MZI without a cladding (and selective spotting of the sensing arm) will potentially outperform an MZI with a cladding for this type of noise.

**Figure 2:**
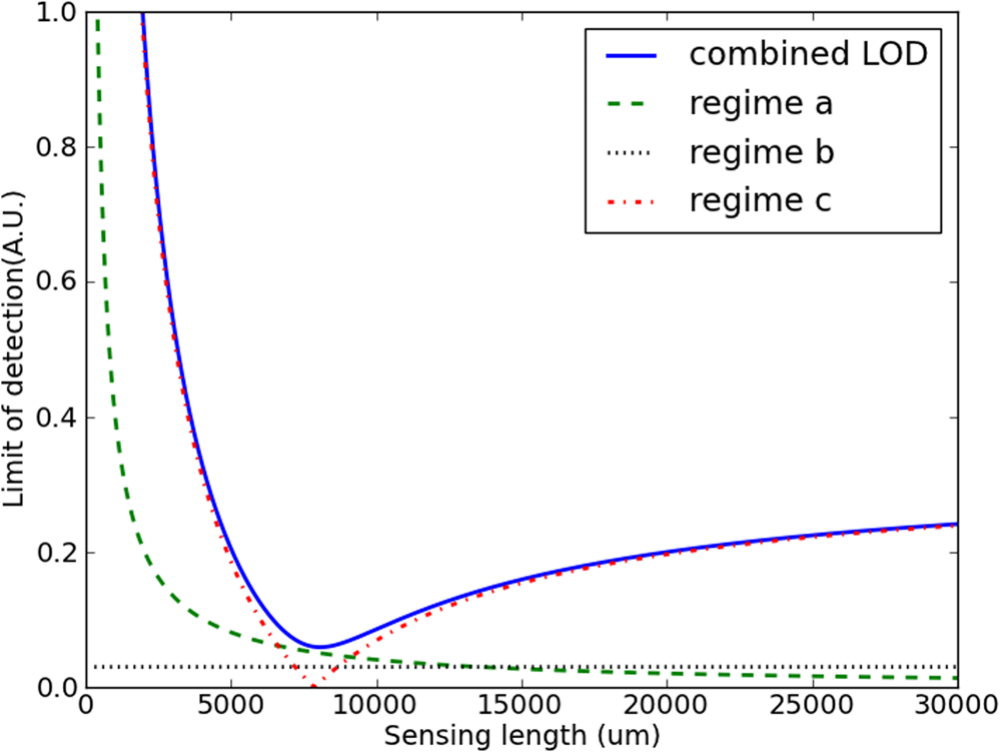
Curves of the three separate regimes, and their combination into a single LOD curve. At various sensor lengths, different regimes can be dominant. The *σ* values are arbitrarily chosen 100.0, 0.03 and 10.0 respectively. The MZI parameters are identical to those of Fig. 1.

In practice, for a generic sensor system, all of these types of noise will apply simultaneously. Combining the *σ*s yields the following equation:

13 $$LO{D}_{full}=\sqrt{{(\frac{3c{\sigma }_{a}}{2\pi \nu {L}_{s}})}^{2}+{(3{\sigma }_{b})}^{2}+{(3{\sigma }_{c}|\frac{{C}_{sT}}{{C}_{rT}}-\frac{{n}_{g,s}}{{n}_{g,r}}\pm \frac{c}{{p}_{\nu }{n}_{g,r}{L}_{s}}|)}^{2}}$$

here, different sources following the same regime can be combined in a single *σ* per regime if necessary. The shape of this curve, and what the best strategy to improve the LOD from a sensor point of view heavily depends on the ratio of the *σ*s. In general, this curve consists of two hyperbolic components with respect to *L*_*s*_ and two constants. For low lengths, these two hyperbolic components will compete for dominance. For longer sensors, the two constants will determine the final LOD. It is possible that a minimum occurs in the intermediate regime. An example of combination of the different regimes is shown in Fig. 2.

This means that the LOD at very high sensing arm lengths is determined by one of these constants, either by regime b or by the constant term in regime c. If the latter is the case, this high-length LOD can be improved by reducing the contrast between both arms, if technologically possible. If regime b is already more of a limiting factor than regime c, reducing the group index contrast will yield little effect.

**Figure 3:**
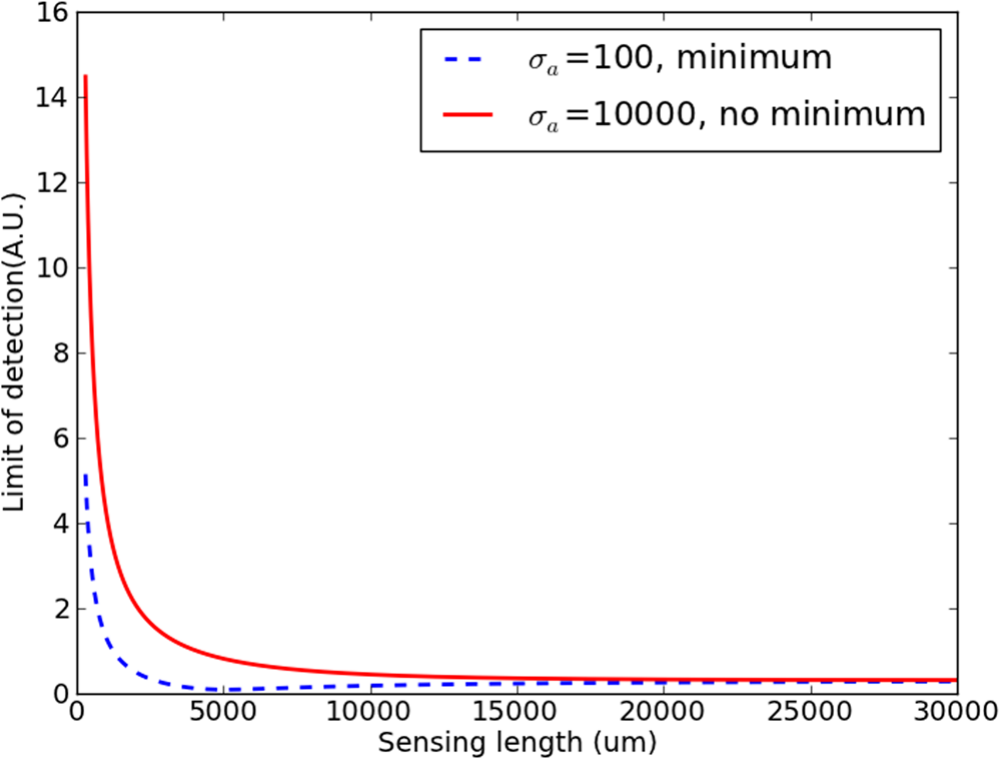
Two possible full LOD curves, the only difference between both being *σ*_*a*_, chosen 10000 and 100. A lower *σ*_*a*_ causes a local minimum to emerge in the dashed curve. In both cases, ${\sigma }_{b}=0.03$ and *σ*_*c*_ = 10. The MZI parameters are identical to those of Fig. 1.

The second important issue is whether the best achievable value is caused by the local minimum found in regime c, or by the value at extremely high lengths. This again depends on the ratio between the various *σ*s and needs to be addressed separately for each sensor system. In general, if regime a is strong with respect to regime c, the minimum will not manifest itself. This implies that a high-noise measurement system might make the minimum inaccessible and the best design strategy in these cases is simply a sensor that is as long as possible (without too much propagation loss, which was ignored in these considerations). Figure 3 illustrates both possible cases.

## Experimental validation

**Figure 4:**
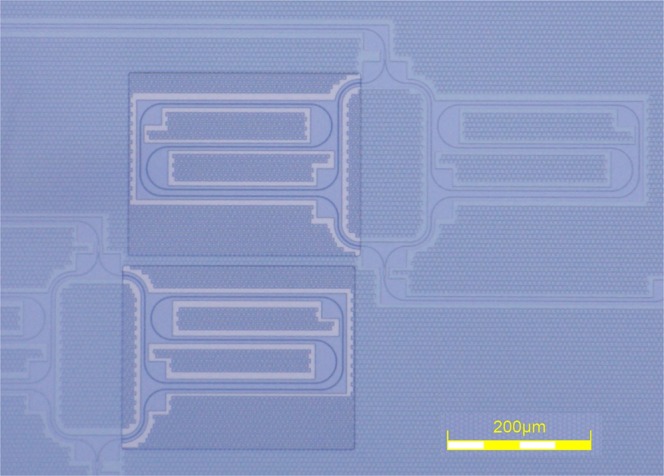
Microscopy image of two Mach-Zehnder interferometers. Both arms are placed in a spiral for efficient space usage. A window in the cladding is opened on the sensing arm.

In this section, we will experimentally validate the achieved behavior through measurements. To this end, various MZI sensors were characterized in experiments similar to those described in^12^. These sensor were fabricated with 220 nm silicon nitride waveguides, with a silicon oxide cladding that was removed for the sensing arms of the Mach-Zehnder interferometers. A microscopy image of such an MZI is shown in Fig. 4. The circuits were designed for and measured in the 850 nm wavelength range. More information on the platform and the fabrication procedure can be found in^16^.

**Figure 5:**
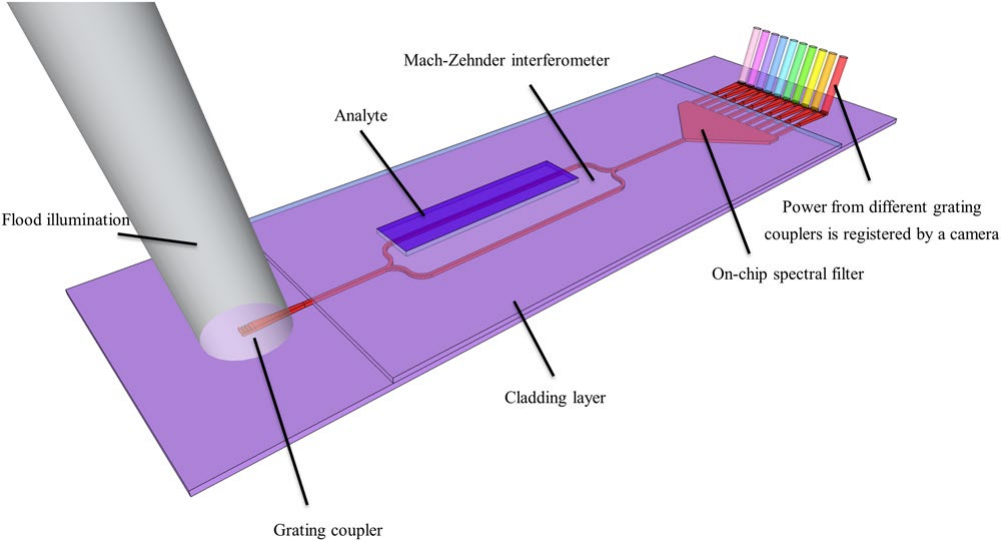
Illustration of the full sensor scheme.

The measured photonic circuits consist of an MZI coupled to an AWG (Arrayed Waveguide Grating, an on-chip spectral filter), read out through a free-space SLED and a camera. Grating couplers are used to coupler light to and from the chip, similar to those described in^13^ The sensor circuit is illustrated through Fig. 5. Due to the cheap readout, this is a system that is particularly suitable for point-of-care applications, and is therefore highly relevant to verify the possibility and method to optimize the limit of detection without increasing the device cost. The sensors were designed to have identical spectral periods of 7 nm, but there was variation (between 5 nm and 10 nm) due to fabrication imperfections (to which the spectral period is highly susceptible). Furthermore, while all sensors were characterized in the same setup, it is likely that conditions slightly varied from experiment to experiment so strictly speaking the *σ* values are not identical, but we will nevertheless assume so. This implies that the obtained results will be indicative rather than qualitative. All measurements were done in a temperature controlled clean room, meaning temperature fluctuations are likely quite small.

To acquire the data, bulk sensing experiments were performed, and as one arm was isolated from the liquid through a cladding, potentially having repercussions for the limit of detection, through the constant factor in the earlier described regime c. In all of these sensors $({L}_{r}{n}_{g,r}-{L}_{s}{n}_{g,s})\,({n}_{g,r}-{n}_{g,s}) > 0$, so it is possible to have a minimum for ${L}_{s}={L}_{r}$. However, because of the low-end read-out mechanism, the hyperbolic component of regime a will likely dominate that of regime c, and therefore the minimum might not occur.

The buffer for all of these experiments was deionized water, the analytes were various water based solutions of either salt or HCl. Because these are very homogeneous mixtures, sample inhomogeneity is likely small. For each experiment, at least four different analytes were used and the sensitivities (and, subsequently, limit of detection) were calculated through a linear fit of the observed peak shifts as a function of the refractive index changes. This method is more elaborately described in^12^.

Two sets of experimental data were collected. The chips were identical, the same buffer and analyte liquids were used, and for data collection the same Thorlabs camera was used for both sets. The only difference between the sets was the light source: the first set was measured with an originally fiberbased Superlum 15 mW SLED (SLED 1), collimated onto the chip in free space. The second set of experiments was measured with a Superlum freespace 20 mW SLED (SLED 2).

**Figure 6:**
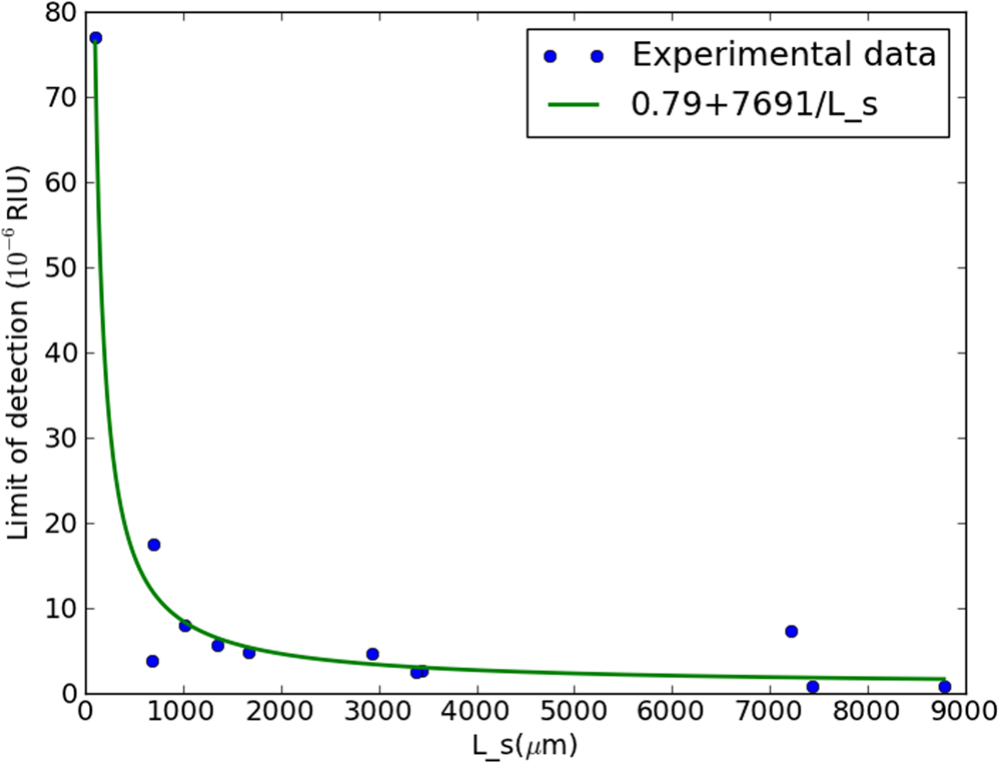
Measured limit of detection as a function of the sensing arm length for various sensors all measured under identical circumstances with SLED 1.

**Figure 7:**
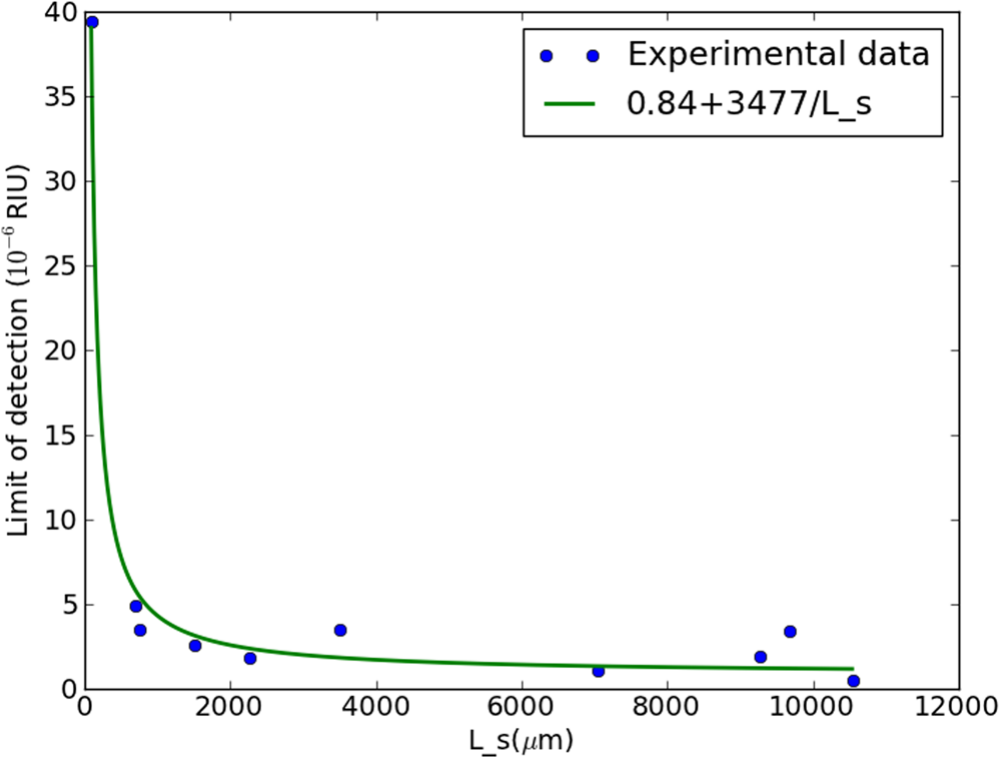
Measured limit of detection as a function of the sensing arm length for various sensors all measured under identical circumstances with SLED 2.

The results are plotted in Figs 6 and 7 respectively. It is immediately apparent that the local minimum does not manifest itself in either dataset. Because of this as well as the limited size of the datasets, we assume one dominant hyperbolic component and one dominant constant factor and fit a curve of a + x/b to both datasets. For both datasets, a relatively well-matching result is obtained, as shown in Figs 6 and 7, apart from a few outliers.

With respect to the dominant regimes: for the hyperbolic component, significantly different fit values are obtained with 1/*L*_*s*_, 7691 and 3477 respectively. This indicates that the light source, the only difference between both sets of experiments, is the determining factor, confirming that regime a is dominant for the hyperbolic component. This is as expected, as the alternative to a dominant regime a for the hyperbolic component is a dominant regime c. The latter is unlikely due to the experiments taking place in a well controlled environment, yielding a low *σ*_*c*_. This furthermore explains why the local minimum is not observed, as this would only emerge through a dominant hyperbolic component of regime c.

The constant terms on the other hand are very similar for both curves with values of respectively 0.79 and 0.84. This is in agreement with the framework, as this term can be determined by a phenomenon of regime b or regime c, neither of which can be affected by the light source, which was the only change between the sets of measurements. Based on these measurements, it is not possible to determine whether regime b or regime c is dominant, as both are expected to be relatively low: the homogeneity of the liquids yields a low *σ*_*b*_, and the well controlled environment yields a low *σ*_*c*_. These sets of measurements illustrate how this framework can be used to optimize the efficiency of a sensing system: for a different LED, the same sensor length can yield a different LOD, apparently suggesting one SLED outperforms the other within this sensor system. However, analysis through the framework shows that both SLEDs yield the same fundamental limit, if the sensing length is chosen sufficiently high. This implies that either of the proposed SLEDs (the most cost-effective or practical) can be used for read-out, without any cost on the LOD, as long as the sensing arm length is sufficiently high. Similar analysis can be performed for any MZI-based sensor platform, opening the path to optimizing efficiency and performance.

## Conclusion

In this work, the different possible limiting factors for the limit of detection on Mach-Zehnder interferometer based refractive index sensors were examined. Particular attention was given to the evolution of the limiting factor for increasing sensing length, as this potentially allows improving the limit of detection purely through sensor design, without complicating the read-out. Three different possible regimes were identified and analyzed, including guidelines for optimizing this sensor type for a given application. The theoretical framework was confirmed through two sets of measurements. This framework opens up the possibility of a more thorough analysis of the limiting factor of any MZI-based sensor system, and as such paves the way to more performant and/or more efficient sensing devices.
